# The coverage of cultured meat in the US and UK traditional media, 2013–2019: drivers, sources, and competing narratives

**DOI:** 10.1007/s10584-020-02813-3

**Published:** 2020-09-02

**Authors:** James Painter, J. Scott Brennen, Silje Kristiansen

**Affiliations:** 1 Reuters Institute for the Study of Journalism, University of Oxford, 13 Norham Gardens, Oxford, OX2 6PS UK; 2 Reuters Institute for the Study of Journalism and Oxford Internet Institute, University of Oxford, 13 Norham Gardens, Oxford, OX2 6PS UK; 3 College of Environmental Science and Forestry, State University of New York, 112 Marshall Hall, 1 Forestry Drive, Syracuse, NY 13210 USA

**Keywords:** Cultured meat, Lab-grown meat, Media coverage, Actor analysis

## Abstract

‘Cultured’ meat has attracted a considerable amount of investor and media interest as an early-stage technology. Despite uncertainties about its future impact, news media may be contributing to promissory discourses, by stressing the potential benefits from cultured meat to the environment, health, animal welfare, and feeding a growing population. The results from a content analysis of 255 articles from 12 US and UK traditional media from 2013 to 2019 show that much of the coverage is prompted by the industry sector, whose representatives are also the most quoted. Positive narratives about cultured meat are much more prominent than cautionary ones. Our findings support previous scholarship on other emerging technologies which concluded that with important variations, media treatments are largely positive.

## Introduction

Cultured, cell-based, cultivated, in vitro, or clean meat^1^ is an emerging technology in which stem or satellite cells are typically taken from the muscle cells of an animal, usually those cells which are responsible for the natural process of repairing a muscle (Datar and Betti 2010; Post 2012; Bhat et al. 2015), in order to produce meat that does not come from slaughtered animals. The end product is sometimes called different names such as ‘lab-grown’ or ‘test tube’ meat. It is part of a wider process called cellular agriculture, which includes production of milk, egg white, and leather from cell cultivation (Stephens et al. 2018: 157).

An indication of the growing interest in this technology is demonstrated by its inclusion in the special report on climate change and land, published by the Intergovernmental Panel on Climate Change (IPCC) in August 2019 (IPCC 2019). Chapter 5 of the report focused on a range of solutions and policies for reducing the greenhouse gas emissions (GHGs) from the land and food sector, of which one section discussed cellular agriculture (5.5.1.6). This section summarized the state of research on cultured meat and suggested that it should be considered as ‘an option for a limited resource world, rather than a mainstream solution’ (IPCC 2019, 5–75).

So while not dismissive of cultured meat, the IPCC report did draw attention to a number of uncertainties about its future production, including its economic feasibility. It cited a recent study suggesting that ‘cultured meat may be even more detrimental than exclusive beef production’ due to its potential heavy energy use (IPCC 2019, 5–76, citing Lynch and Pierrehumbert 2019). It also stated that the market for cellular meat products was ‘largely unknown’, and concluded that ‘its actual contribution to climate change mitigation and food security is largely uncertain and challenges are not negligible’ (IPCC 2019, 5–76).

The IPCC assessment highlighting the uncertainties surrounding cultured meat came almost exactly 6 years after the public arrival of the first ‘lab-grown’ beef burger, which was launched at a venue in West London on 5 August 2013 with considerable optimism. It was the product of established biomedical technologies (such as tissue engineering) being applied to a new application (food production). The new product—a five-ounce burger, created by Professor Mark Post from the University of Maastricht based on 20,000 strips of muscle cell—was paraded in front of dozens of journalists and the image of it beamed live around the world by several television channels. In a video presented at the event, Google’s co-founder, Sergey Brin, who had invested a reported US$250,000 in research to develop the burger, stressed what he called ‘the potential transformative nature of the technology’^2^ Since the launch, interest from the media and investors in the cultured meat industry has continued, and, in the last couple of years, grown significantly.

In this study, we first place the discussion of cultured meat in the context of media treatments of other emerging technologies. We then summarize some of the key aspects of the development of cultured meat, including its potential benefits and uncertainties, and then review the previous studies of its coverage in the media. The main section consists of a detailed assessment of 6 years’ coverage in US and UK traditional media from 2103 to 2019, examining the news pegs that prompt coverage, the sources quoted, the dominant narratives, and the general sentiment found in the 255 articles we analysed.

In this way, we make a substantial update of previous research in this field and seek answers to whether technology companies and investors have continued to dominate the way the discussion about cultured meat takes place in part of the public sphere. In addition, we assess whether the media may be responsible for a form of ‘techno-optimism’, which goes beyond the current scientific understanding of the prospects for cultured meat, and the uncertainties surrounding it.

### The media and emerging technologies

Cultured meat has joined a set of new and emerging technologies that the media often describe as meaningful solutions to a range of pressing public problems, even as their promise remains largely unproven. Much of the research on news coverage has focused on nanotechnology (Scheufele and Lewenstein 2005; Donk et al. 2012; Dudo et al. 2011), biotechnology (Nisbet and Lewenstein 2002; Caulfield 2004; Priest 2008), or communication technologies (Arceneaux and Schmitz Weiss 2010).

While some have suggested that coverage of new technologies evolves across the issue cycle, there remains some ambiguity in the general sentiment of coverage of emerging technologies. Metag and Marcinkowski observe that a number of studies in the 1980s and 1990s found a persistent ‘negativity bias’ in coverage of new technologies (2014, p. 464). This negativity bias was seen in technologies ranging from biotechnology (Bauer et al. 2001; Marks et al. 2002) to the telegraph (Czitrom 1982). More recently, Vilella-Vila and Costa-Font found that British and Spanish media coverage of GMOs emphasized ‘its risks, framing the reality of GM food as a highly controversial issue’ (2008, p. 2104).

In contrast, other scholars have more recently found that for many emerging technologies ‘coverage is mainly positive and focuses on the medical, scientific or economic benefits’ (Metag and Macinkowski 2014, p. 475). Nisbet and Lewenstein (2002), for example, observe that American coverage of biotechnology has been ‘overwhelmingly positive’, and has often emphasized frames of ‘scientific progress and economic prospect’ (p. 2). Similarly, Kitizinger and Williams (2005, p. 379) noted a notable bias toward the future in media discussion of stem cells, such that ‘the real battleground is about the plausibility of diverse visions of utopia and dystopia and about who can claim the authority (in terms of both morality and expertise) to produce a credible version of the future.’

Brennen et al. (2018, p.1) found that the recent UK media treatment of artificial intelligence (AI) was also generally positive, ‘portraying AI as a relevant and competent solution to a range of public problems [….] with little acknowledgement of on-going debates concerning AI’s potential effects.’ Arceneaux and Schmitz Weiss (2010) observed that early coverage of Twitter, like that of personal computers (Cogan 2005), was consistently positive, heralding everything from its ability to ‘make valuable business contacts or find jobs’ (p. 9) to facilitating political mobilization and ‘access to political information’ as politicians embraced it.

There is no simple linear relationship between the representations of emerging technologies in the media and audience reaction, but research has broadly confirmed that media depictions can affect public attitudes toward them (Nisbet and Lewenstein 2002; Scheufele and Lewenstein 2005; Anderson et al. 2011). Lee et al. (2005) argue that public sentiment about new technologies, such as nanotechnology, is driven by a combination of ‘knowledge and affect’: technical understanding or knowledge as well as emotional frames and stories about technologies. In addition to providing information about new technologies, media can supply or influence the ‘emotional heuristics’ which laypersons use to evaluate new technologies (Lee, Scheufele and Lewenstein 2005, p. 262). That is to say, ‘media frames provide audiences with cognitive shortcuts or heuristics for efficiently processing new information, especially for issues that audience members are not very familiar with’ (Scheufele and Lewenstein 2005, p. 661).

### The development of cultured meat

In the early phase from 2000 onwards, the research into cultured meat took place largely within a biomedical academic context, but this changed after the lab-grown burger launch event in 2013. Several start-ups announced their arrival, supported by different strands of venture capital (Stephens et al. 2019). Particularly since 2015, innovation and private investment in the cultured meat sector has increased significantly and quickly, albeit from a very low base (Froggatt and Wellesley 2019). According to a June 2019 report from the Good Food Institute (GFI), a lobby group based in Washington DC on behalf of alternatives to conventional meat, the amount of investment in cultured meat companies has grown from about US$2m in 2015/6, when the US company Memphis Meats received seed funding from IndieBio, to nearly US$50m in 2018 involving 12 companies in 14 deals (Cameron and O’Neill 2019). This figure was considerably less than the US$600m invested in plant-based alternatives in 2018 (ibid., p.20), but since 2018 there have been a number of other significant investments in the sector including a successful second round of funding of more than US$160m in Memphis Meats. Several high-profile individuals and companies who had already invested in Memphis Meats, such as Bill Gates, Richard Branson, Cargill, and the US meat-packing company, Tyson Foods, took part.^3^

Most of the investment has come from private sources, but according to media reports, the Chinese government and government-funded agencies in Singapore and Japan have also invested in the sector (Stephens et al. 2018). According to the GFI, in 2018 there were a total of 15 funded companies, across three continents, but market analysis suggests that since then the number has risen to at least 30.^4^ These companies are developing different types of alternatives to meat (beef, chicken, turkey, and duck) and fish, as well as leather replacement, all based on cellular technology. The regulatory framework, involving aspects of labelling and food safety, is also the focus of growing attention both in the USA and Europe (Cameron and O’Neill 2019).

### Potential benefits and uncertainties

One of the main arguments used by cultured meat advocates is that it could have a positive impact on the environment according to several metrics, including reduced water use and water pollution, fewer greenhouse gas (GHG) emissions, and less land use compared with conventional livestock meat production. One of the first studies to be carried out suggested that cultured meat has 78–96% lower greenhouse gas emissions, 99% less land use, and 82–96% less water use than the conventional breeding, raising, and slaughtering of cattle or other livestock (Tuomisto and de Mattos 2011).

However, more recent work—including by Tuomisto (2018)—has been more cautious in their projections of GHG emissions from cellular agriculture, particularly when compared with unprocessed plant-based products (e.g. peas, beans) and, in some scenarios, poultry and pork (Mattick et al. 2015b). Researchers in this field point to the need for more high-quality, peer-reviewed life cycle assessments (LCAs) carried out on specific production processes to determine the trade-offs with respect to livestock rearing (Lynch and Pierrehumbert 2019; Mattick et al. 2015a). Lynch and Pierrehumbert (2019) concluded that it is not yet clear if cultured meat will provide a more climatically sustainable alternative to conventional beef production. They stress that this will depend on the levels of decarbonized energy generation (i.e. renewables) used in cultured meat production, and on the specific environmental footprints of production. However, their modelling suggests that if the CO_2_ footprint of cultured meat is sufficiently low, it will prove climatically superior to all forms of conventional beef production into the long term.

Cultured meat is often described in media and academic discussions as a more humane or ethical way to produce animal flesh for human consumption as it reduces animal suffering (Hopkins and Dacey 2008; Schaefer and Savulescu 2014). It is also frequently reported that cultured meat could represent a healthier option than conventional meat, in part because of the more sterile production environment, and in part because of the heavy use of antibiotics in meat-producing animals as a source of antibiotic-resistant bacteria (see for example, Zaraska 2016).

A fourth main area of potential benefit is the possibility that cultured meat can help feed a growing world population, where demand for meat is projected to increase substantially in the coming decades and to put more pressure on land use. Public consumption of meat is widespread in high-income countries, and relatively stable, but is rising rapidly in many regions of the world, and particularly in China (ILRI 2019). Other, less discussed, potential benefits include the promise of a better ‘taste experience’, the possibility of considerably higher returns per animal than traditional farming, reduction of food waste, and new opportunities for those farmers using traditional native breeds of livestock (Sexton et al. 2019; Stephens et al. 2018).

One major area of uncertainty is the variety of technical challenges involved in producing different types of cellular meat products, which include the cell source, culture media, mimicking the in vivo myogenesis environment, animal-derived and synthetic materials for the scaffold and the media, bioprocessing for commercial-scale production, and safety concerns (Hocquette 2016; Stephens et al. 2018; Dolgin 2019).

The technical challenges are one determinant of how soon a cultured meat product could come to market, and if so, whether it will be affordable to all socio-economic classes, and not just an elite high-income consumer (Pluhar 2010). A considerable amount of discussion also surrounds the regulatory frameworks in the USA, the EU, and elsewhere, which could constrain the ability to market cultured meat (Froggatt and Wellesley 2019; Schneider 2013; Stephens et al. 2018).

Finally, major uncertainty surrounds likely public attitudes to consumption of cultured meat, which could be a major barrier to its success (Sharma et al. 2015). Existing studies from different countries show a wide range of opinions from very supportive to very negative (Stephens et al. 2018). Bryant and Barnett (2018) highlighted demographic variations and different factors influencing acceptance, while the same authors (Bryant and Barnett 2019) found greater consumer acceptance when the product was described as ‘clean meat’ or ‘animal free’ meat compared with ‘lab-grown’ meat. But public acceptance of cultured meat is hard to gauge accurately when firstly, cultured meat is a technically feasible but still hypothetical consumer product (Broad 2020), and secondly the survey work is not based on consumer sampling of the actual product.

### The media and cultured meat

There have been only a handful of studies of how traditional media have covered the specific issue of cultured meat. One study of US (including local print) and European newspapers from 2005 to 2011 carried out a qualitative analysis of a small sample of 34 articles (Goodwin and Shoulders 2013). It identified common themes, including the benefits in four main areas (environmental, animal welfare, food security, and human health), the current problems with livestock production, the history and processes behind cultured meat, and doubts about its future, including possible consumer rejection. The study identified key sources (restaurant owners, academics, and other researchers, amongst others) quoted in the media, and observed that ‘very few sources opposed the production and few represented the agricultural industry’ (p.449).

A second study (Dilworth and McGregor 2015) identified several common ethical discourses about clean meat in the academic literature from 2002 to 2013 (such as environmental sustainability and animal welfare), and compared them with their presence in Australian print media from January 2005 to December 2013. One of its main conclusions was that discourses critical of cultured meat’s wider socio-cultural implications have received little media attention.

A third study (Hopkins 2015) examined the coverage in the UK, US, and Canadian media of the launch of the lab-based burger in August 2013. Although aspects of the methodology and media selection are unclear, the research found that the benefits highlighted in the media were the reduced environmental impact and land use, reduced harm to livestock animals, and human health advantages. Importantly, the study noted that there was no mention at all of the reaction of any type of meat producers but rather the reaction of vegetarians was prominently featured. The media were guilty, the author argued, of creating a skewed impression of the importance of vegetarians, at the expense of the more important demographics of meat eaters and ‘the empirical psychology of mainstream consumers’ (ibid. p. 264).

Sexton et al. (2019) identified five ‘promissory’ narratives about alternative proteins (APs) including cultured meat, and then relied in part on articles from the traditional media in 2013–2014 and 2017–2018 to pick out the counter narratives produced by the livestock industry to dispute them. Broad (2020) also relied partly on media analysis (combined with interview work and participant observation) to identify two common metaphors found in the discourse of AP advocates. The first is that ‘meat is made’, by which AP advocates ‘argue that meat need not be defined by its animal origins, but rather characterized by a set of tastes and textures, composed at the molecular level through a combination of enzymes, amino acids, and, most importantly, proteins’ (p. 6). The second series of metaphors is around the primacy of the ‘market’ which ‘highlight the role of innovation, investment, industry collaboration, behavioral economics, and marketing as drivers of that process’ (p. 11).

Drawing on the discussion above, our research is aimed at giving insights into these four research questions:

1. Who or what are the most common news pegs for prompting the mainstream media to cover cultured meat issues?
2. Who is being given space by the media to discuss cultured meat?
3. What are the most common promissory and cautionary narratives used to shape the discussion?
4. To what extent do the news articles show positive, neutral/balanced, or negative sentiments toward cultured meat?

## Method and research design

In order to seek answers to these questions, we analysed online and print versions of twelve traditional media organisations, divided equally between the USA and UK, in the period from 1 January 2013 (to include the year when the first lab-grown burger was launched) to the end of March 2019. Despite the many changes to media landscapes, traditional news outlets remain a key space for, and influence on, public discussion, in terms of setting agendas and focusing public interest, particularly in those areas in which audiences do not possess direct knowledge (Happer and Philo 2013); most are more trusted and used than more recent arrivals and social media (Newman et al. 2019).

The six from the USA were the Boston Globe, the Los Angeles Times, the New York Times (NYT), USA Today, the Wall Street Journal (WSJ), and the Washington Post, and the six from the UK the Financial Times (FT), the Guardian/Observer, the i/Independent, the Mail, the Telegraph, and the Times/Sunday Times. However, the division between US and UK titles is slightly misleading as in recent years, the Guardian, Mail, FT, and Telegraph have followed an active policy of attracting readers in the USA and around the world. We chose the USA and the UK because of the location there of many important media titles with a worldwide reach and influence amongst policy makers (O’Neill et al. 2015), and of investors in the sector, legislative initiatives, and NGOs, think tanks, and pro-industry bodies.

The titles were chosen to represent a variety of political leanings and editorial priorities. Of the six UK titles chosen, five appear in the list of the 16 most read print and online sites compiled by the Reuters Institute in 2019 (Newman et al. 2019, p. 69). The Financial Times is the one not to appear, but it is well-known as a paper and website of record for business coverage in the UK and beyond. However, the paper does appear second in the list of most trusted brands in the UK, above the other five titles which also appear. Of the six US titles, the Washington Post, NYT, USA Today, and city titles (such as the Boston Globe) feature in the list of the 16 most read print and online sites. The WSJ does not appear in this list, but it comes second in the list of the most trusted brands (ibid., p. 118).

We searched for articles in the Factiva data base using the Boolean search-string: ‘Meat AND (cultured OR lab-grown OR in vitro)’. ‘Cultured’ and ‘in vitro’ were chosen as they were the terms used in the previous literature on mainstream media analysis (Goodwin and Shoulders 2013; Dilworth and McGregor 2015; Hopkins 2015), and ‘lab-grown’ was included as journalists have tended to adopt the phrase (Broad 2020). We found that the addition of the word ‘cell-based’ hardly added to the number of results, and the word ‘clean’ included many articles way beyond the focus of this study. Initial results gave 607 articles for the UK six titles, which were reduced to 169 after filtering for irrelevant articles, repeats, letters, travel guides, and trails, and 304 articles for five of the US titles, which was reduced to 75 after filtering. For the Los Angeles Times, we used the same search terms in ProQuest Newstream backed up by a Google News search, which resulted in a total of 11 LA Times articles after screening. This gave a total sample size of 255 articles. Cultured meat or a variant of it had to appear at least once in the article for it to be coded.

The codebook was initially developed deductively, based on previous studies of media coverage of scientific issues for news pegs and sources (Brennen et al. 2018), and of cultured meat for the common positive and negative narratives discussed in the literature (Sexton et al. 2019; Stephens et al. 2018; WEF 2019). After initial testing, the codebook was then inductively re-designed to incorporate more, or different, positive (‘promissory’) and negative (‘cautionary’ or ‘counter’) narratives commonly found in the media articles. The articles were also coded for type of article (news, feature, opinion), news peg or prompt (such as product release, academic study, NGO report), the main sources quoted (such as company representatives, academics/scientists, farmers, or NGOs), and finally for the dominant sentiment divided into three categories: rather negative/oppositional tone; neutral or balanced (including having no stance, or when pro and con arguments are included to a significant degree); rather positive/promotional tone.

To measure inter-coder reliability, we applied Cohen’s Kappa statistic, which is considered to be a more robust method than a simple percentage agreement calculation, as it takes into account the possibility of inter-coder agreement occurring by chance. Any score < 0.6 is considered inadequate (McHugh 2012). In this study, the initial inter-coder reliability score based on a sample of 25 of the articles was < 0.6 for 10 of the 69 variables coded. These ten variables were then discussed in detail by the two coders, and the codebook refined. A further test of ten articles gave a score of < 0.6 for only three of the variables. For each of these three variables, the result was 0.47, but this was due to only one discrepancy between the two coders in a dominant sequence of zero coding, a result often given by using Cohen’s Kappa.

## Results and discussion

### Volume of coverage

**Fig. 1:**
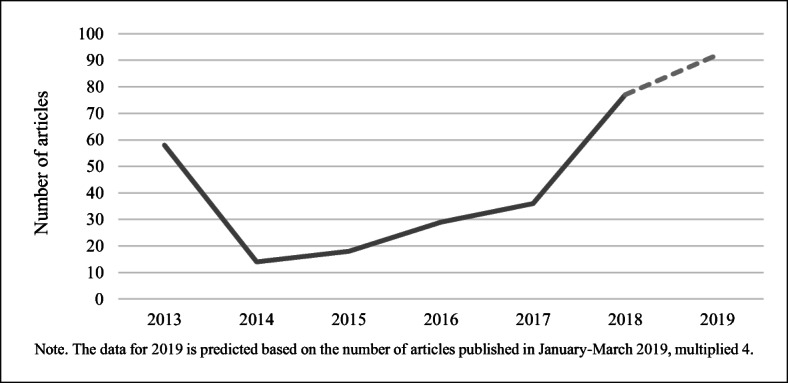
Volume of coverage in the US and UK media, by year, 2013–2019

By way of context, we first plotted the amount of media interest in the topic of cultured meat over the 6-year period. Figure 1 shows a peak in coverage in 2013, which was largely driven by the launch of the first lab-grown burger in August, followed by a sharp decline afterwards and then a slow growth of interest in 2015–2017. 2018 showed another peak, this time largely driven by an increase in the reporting from the WSJ and Washington Post, prompted often by new investment and the debate over labelling. The figure for the first three months of 2019 was 23 articles (21 in the UK titles, 2 in the US titles), and the dotted line represents a projection based on the same quarterly rate.

The total sample of 255 articles was divided between 169 in the UK and 86 in the USA; the higher level of interest in the UK may be in part explained by the greater amount of attention given to climate change in the UK media compared with that of the USA (Schmidt et al. 2013), as the cultured meat discussion is often framed as a part of the solution to environmental problems, including climate change.

**Fig. 2:**
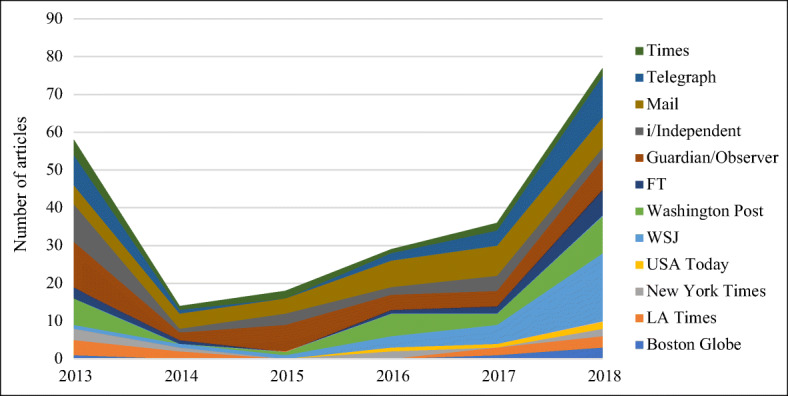
Volume of coverage in the USA and UK, by media title and year, 2013–2018

Figure 2 shows the media coverage broken down by year and media outlet until the end of 2018. For the UK, the Guardian and the Mail had the most coverage sharing about half the number of articles, the Times and the FT the least. In the USA, the WSJ had the most coverage at 35% (29 articles),^5^ followed by the Washington Post at 32% (27), and the other four titles notably lower down. The high volume of coverage in the WSJ was probably a reflection of the amount of US investor interest in the sector, particularly starting in 2017 (see Fig. 2). In contrast, the FT had the equal lowest number of articles in the UK sample at 9% (15).

### News pegs

We defined a news peg as what makes a story timely or newsworthy, and we assessed this by looking at the headline or first three paragraphs of each article. Forty-three percent (110) of the sample had no strong news peg, compared with 57% (145) that did. The overwhelming majority of those with a strong news peg were prompted by an industry source (75%, 83), whether this was the announcement of a new product, new research, a new investment or business decision, a conference/event/speech, or an interview with a company representative.

**Fig. 3:**
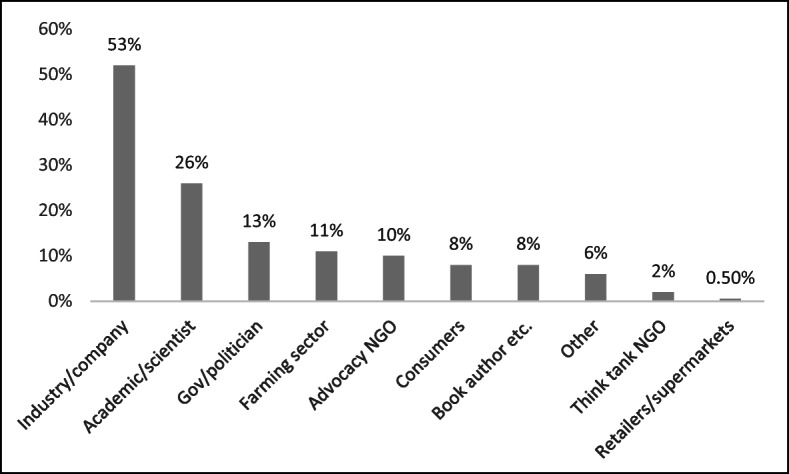
Presence of different types of actors

This category included such pro-industry groups as the Good Food Institute, and the San Francisco–based Cellular Agricultural Society (CAS). Industry sources were followed by ‘Other’ (which included banks and the finance sector) much lower down at 8% (9), government sources at 7% (8), academic research at 6% (7), and NGOs at 4% (4). It should not be surprising that industry sources were the main prompt for journalistic stories, given the nature of coverage of an emerging technology, but it is noteworthy that such a *high* proportion of articles were industry-led, and that there was a much less even spread of actors in the news prompts compared with the distribution of quoted actors shown in Fig. 3.

### Sources quoted

Figure 3 shows the distribution of actors who were quoted either directly or indirectly (such as ‘Actor X said that’) in an article which were either directly relevant to the issue of cultured meat or were more broadly relevant in the context of an article that was mostly about cultured meat. As we can see, representatives of the industry or company sector appeared in over half the articles, followed by academics or scientists, and governments or politicians. The industry sector was more over-represented than the percentage figures suggest; in many articles, either the same representative was quoted several times in the same article or several different representatives were present, compared with other sectors. A total of 222 industry representatives were quoted in the articles, compared with the second highest number, academics or scientists at 91.

The overlap between academia and industry was particularly marked, and the distribution of the percentages may have changed between industry and academic sources if we had coded Dr. Mark Post from the University of Maastricht and others like him as academics. However, most of the articles quoted Dr. Post in the context of his involvement with the company Mosa Meat. Likewise, Gabor Forgacs, Professor of Biological Physics at the University of Missouri-Columbia, and scientific founder of Organovo, and Nicholas Genovese, a university researcher and Chief Scientific Officer of Memphis Meats, were both coded as ‘industry’.

Although academics and scientists (without strong industry affiliations) had the second largest presence in over a quarter of articles (26%, 66), fewer than 5 of the 40 individuals cited were biologists or engineers pursuing technical research related to cultured meat in academia. The rest included a wide range of social scientists, philosophers, nutritionists, communication scholars, and historians. Some of these researchers have been explicitly studying the social, political, economic, or environmental implications of cultured meat. Others, such as Richard Dawkins or Yuval Harari, have pursued research broadly relevant to the topic. Of these academics, Hanni Rützler appeared most frequently. She was one of the two experts invited to taste a sample of cultured meat in 2013.

We did not systematically code all the references to academic papers in our sample. However, on three occasions, the early work by Hanna Tuomisto^6^ (Tuomisto and de Mattos 2011) was specifically referred to, quoting the advantages of cultured meat expressed as percentage drops in GHGs and water and land use. Both a Washington Post article of 6 August 2013 and a NYT article of 14 May 2013 included links to her 2011 research. A MailOnline piece on 2 May 2018 (based on Reuters) entitled ‘Would YOU eat meat grown in a lab?’ again quoted her 2011 paper giving percentage reductions. In this last instance, suitable caveats were included about the imprecision of such studies.

Besides Rützler and Tuomisto, most academics only appear in a very small number of articles, indicating both that the larger field of research on cultured meat is still being established and that journalists are casting a wide net in looking for academic sources of commentary.

Government, government bodies, and politicians appeared the third most quoted in 13% (33) of the articles. This was not a reflection of a significant political debate around the issue, but more often of the US regulatory bodies such as the US Department of Agriculture (USDA) and the US Food and Drug Administration (FDA) taking an interest in legislation surrounding the industry. The farming, livestock, and dairy sectors were relatively neglected, as they were quoted only in 11% (27) of the articles. This is perhaps prima facie surprising, given how much their interests could be affected. It is also noteworthy that their presence was almost equally distributed between the UK sample (14 times) and the US sample (13 times) despite the much larger UK sample, which was in part a reflection of the greater presence of the US Cattlemen’s Association, the National Cattlemen’s Beef Association (NCBA), and state-level cattle sector groups in the media (mentioned in 11 articles).

All but one of the quotes from the US cattle groups appeared from early 2018 onwards. The context is that this was the period in which the USDA, FDA, and lobby groups were debating the issue of labelling cultured meat products and other regulatory measures. In August 2018, Missouri became the first state in the USA to pass legislation prohibiting food makers from using the word ‘meat’ to refer to anything other than animal flesh. Arkansas has passed similar legislation, and other states are considering or have passed similar laws.

In a feature article, the Washington Post (24 February 2019) pointed out that ‘Fighting fake meat <was> the NCBA’s top priority for 2019’. The article included several quotes from the then president of the NCBA, Kevin Kester, including his view that the US consumers would choose ‘real food’. Kester said that ‘Personally, I’d choose a tasty, traditionally produced rib-eye steak over a tofu burger or something out of a petri dish every single day of the week’.

The articles published throughout 2018 both in the US and UK samples gave considerable space to the cattle industry’s views that the meat label should only be allowed on products that come from slaughtered animals. Jim Dinklage, the president of the Independent Cattlemen of Nebraska, was typical in saying that ‘the word meat, to me, should mean a product from a live animal’ (NYT, 11 February, 2019). In our sample, we also found several mentions of the US Cattlemen’s Association’s argument that the term ‘meat’ would confuse consumers, and their preference for other terms like ‘cultured tissue’ or ‘fake meat’.

It is interesting to note here that these voices only started to appear in the context of debates about labelling regulation, and were not a consistent voice throughout the whole period. It is also noteworthy that in the UK, where the issue of regulation has not arisen strongly in the public sphere, the National Farmers' Union was only quoted once in the context of their February 2019 report on the future of farming (which mentioned ‘in vitro meat’), even though several articles mentioned the possible long-term threat to their industry and livelihoods posed by the development of cultured meat.

### Positive and negative narratives

**Fig. 4:**
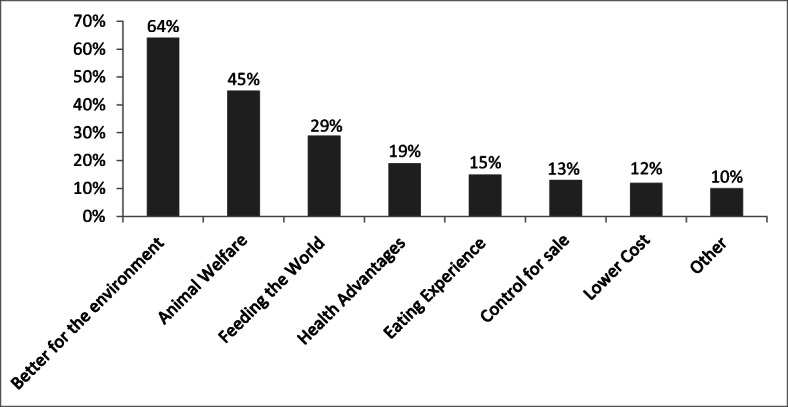
Presence of positive narratives in articles

**Fig. 5:**
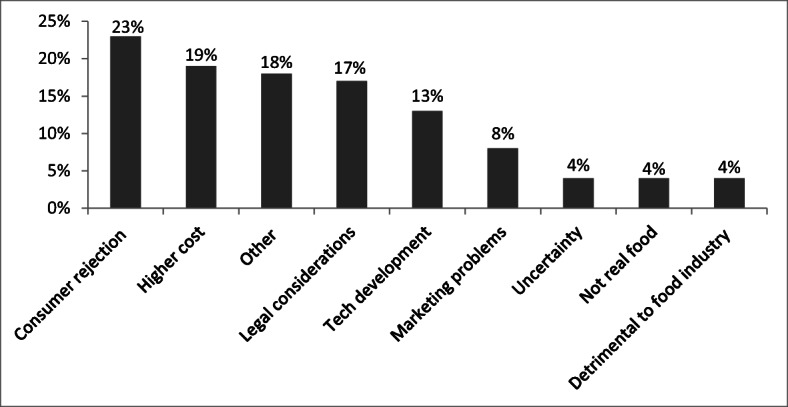
Presence of negative narratives in articles

Figures 4 and 5 show the percentage figures for how the different positive and negative/counter narratives about cultured meat were present in the sample of articles. The ‘better for the environment’ narrative was the most present in 64% (163) of the articles. This included mention of lower GHGs, less land and water use compared with conventional farming, or less deforestation and biodiversity loss. These narratives took several forms, including a journalist giving a positive take on the cultured meat industry’s ambitious aims as in ‘<the world’s first lab-grown steak> marks a significant step forward for a nascent industry that aims to provide people with real meat without the huge environmental impact and welfare problems of intensive livestock production’ (Guardian 14 December 2018); or the frequent direct quoting of industry lobby groups as in ‘Impossible Foods say its burger cuts GHGs by 87%’ (Guardian, 30 April 2018).

The second most common positive narrative was ‘animal welfare’ at 45% (115), which included any mention of the phrase ‘slaughter-free’. It is significant that nearly half of all our sample expressed this sentiment in some form, as for example in the Daily Telegraph (31 January 2019, ‘Others simply believe animal-free meat represents the next big thing for consumers, concerned about poor conditions and cruelty in factory farms’. The third most present narrative was ‘feeding the world’ at 29% (74) of the articles, which included any mention of improved food security, followed by health advantages at 19% (48) such as ‘Scientists and businesses working full steam to produce lab-created meat claim it will be healthier than conventional meat’, found in the Washington Post, 2 May 2016.

Our findings from the media analysis about positive narratives do roughly match the five promissory narratives identified by Sexton et al. (2019), which they based on an empirical analysis of the statements by 12 leading Alternative Protein stakeholders, namely ‘Healthier bodies’, ‘Feeding the world’, ‘Good for animals and the environment’, ‘Control for sale’, and ‘Tastes like animal’. However, it is clear that ‘good for animals and the environment’ were two separate narratives present in the media sample, even though the ‘better for the environment narrative’ was found in 67% of the articles at the same time as one or both of the next two most frequent positive narratives. This does suggest that journalists often bundled together the three main perceived advantages of cultured meat.

The Sexton et al. (2019) ‘control for sale’ narrative includes the uncleanliness of conventional farming and the use of hormones, which are mentioned in our sample at 13% (33); their ‘tastes like animal’ narrative is roughly the same as our ‘eating experience’, which again features quite strongly in the sample at 15% (38). This is mostly evidenced by claims from companies like this one from Aleph Farms quoted in the WSJ (11 December 2018): ‘Israeli-based Aleph Farms says it’s figured out how to create the structure of real beef from animal cells in a petri dish — so that it actually feels like you’re biting into a piece of meat, rather than just tasting it.’ However, it is worth pointing out that other promissory narratives were present in our sample too, particularly the industry claim of lower costs (12%, 31) and the ‘other’ category (10%, 26), which included the possibility of increased consumer demand, and traditional meat production and consumption becoming generally unsustainable or unethical.

‘Consumer rejection’ was the most present counter narrative in 23% (59) of the articles, followed by higher cost (19%, 48), ‘other’ at 18% (46), and legal considerations such as the labelling of cultured meat at 17% (43). The first category took several forms, including rejection on the grounds of price, taste, naturalness, safety, and reluctance to diverge from traditional meat. The ‘other’ category here included the (potentially) negative nutritional impact of cultured meat, the distraction from necessary changes to a plant-based diet, the argument that cultured meat is not vegan, its time-consuming or labour-intensive production processes, and its mass production being many years away. An example of this category would be the UK Vegetarian Society being quoted in the Boston Globe of 2 July 2017, asking the question: ‘Why bother to create artificial meat when a balanced vegetarian diet is delicious, nutritious, and sustainable?’

Many of the articles discussed aspects of the ‘eating experience’ of cultured meat, both real (as performed by two food critics at the launch of the lab-grown burger in August 2013) and anticipated or imagined. Indeed, several of the articles ran headlines encouraging readers (as consumers) to imagine trying cultured meat such as ‘Would you eat…?’ or ‘Could you tell the difference…?’ The ‘consumer rejection’ we mapped in our sample thus included different elements: (i) anticipated consumer reaction, based on grounds of taste often described as the ‘Yuk’ or ‘ick factor’ (as in the headline in the Washington Post of 3 May, 2016, ‘Meatballs made in a lab: Ick or slick?’; (ii) the journalistic quoting of consumer survey work (such as ‘in a recent survey only a quarter of people found the idea “very” or even “somewhat appealing”’, as reported in the Guardian 16 March, 2019); and (iii) the ‘not real food’ argument put forward by specific interest groups such as the cattlemen’s associations (such as reporting that ‘If it didn’t come from an animal, it isn’t meat in Missouri’ in the Daily Mail, 29 August 2018).

The anticipation of consumer rejection was one of the counter narratives identified by Sexton et al. (2019) put forward by the livestock sector, as were the uncertainties around the speed and development of the technology to produce cultured meat products. This was found in 13% (33) of the articles. The characterisation of APs as artificial or synthetic compared with conventional animal foods was another counter narrative, which was part of the narratives found in our ‘not real food’ category. This was present in just 4% (10) of the articles, in contrast to another counter narrative of legal considerations (including general regulatory uncertainty, contested or absent labelling, or no regulatory oversight), present in 13% (33) of the articles. So we can say that the media narratives did include, and match, some of the arguments put forward by the US livestock sector, but others such as higher costs (19%, 48) and the ‘other’ category mentioned above were strongly present too.

Finally, it is of interest that the three counter narratives highlighted by the IPCC in its 2019 report (IPCC 2019), namely marketing problems, economic feasibility, and uncertainty, were represented much less than the three main positive narratives. The uncertainty narrative (which we interpreted as uncertainty or insufficient knowledge over environmental impact, cost, nutritional or health advantages, or amount of energy needed) appeared in only 4% of the articles.

### Sentiment analysis

A positive or promotional tone was found in nearly half of the articles (49%, 125) compared with just 3% (8) which showed a negative or oppositional tone (the remainder was neutral or balanced). The overwhelmingly positive treatment of cultured meat had various manifestations:

- During the extensive media coverage of the launch of the lab-grown burger in August 2013, most of the reporting was balanced or neutral, particularly in the coverage of the taste experience which was largely along the lines of ‘the jury is out’. However, a number of editorials and opinion pieces at the time were overwhelmingly positive about its general prospects (in the Boston Globe, NYT, Independent, Times, Telegraph and Guardian).
- In the commentary, editorial, or opinion pieces, which represented about 14% (36) of our sample, the authors were overwhelmingly in favour; they included commentators in the UK from across the political spectrum such as the right-wing Jeremy Clarkson in the Times on 29 November 2015, and the left-wing George Monbiot in the Guardian on 8 June 2018.
- There were several examples of very positive treatments of product launches. One such example published in the Telegraph on 15 March 2017 gave an uncritical report of the first ‘test-tube chicken meat’ launched by Memphis Meats. The article described the product as a ‘huge step forward for the ‘clean meat’ movement’, included unnamed scientists as believing that ‘cultured meat will eventually entirely replace raising animals and that future generations will deem eating animals as unthinkable’, and quoted a representative of the pro-industry GFI as saying the taste of the ‘chicken’ was ‘superb and tender’.

Sentiment did not vary much over time. The year with the highest percentage of positive sentiment was 2016 (69%, 176), and the lowest was 2015 (29%, 74). However, the remaining 5 years (2013, 2014, 2017, 2018, and 2019) fell between 40 and 52%. The US sample revealed only one article with negative sentiment, compared with seven in the UK sample. The UK newspapers showed marginally more positive sentiment (50% of articles, 128) than the US ones (41%, 105).

## Conclusions

In answer to our four research questions, it is clear that an overwhelming proportion of the coverage in our sample is often prompted by the industry sector, whose representatives are also the most quoted. Positive narratives about cultured meat are much more prominent than cautionary ones, and the overwhelming majority of articles show a positive sentiment. So we do find evidence for a ‘prematurely optimistic discourse’ and ‘an abundance of […] aspiration rhetoric (fueled largely by corporate and media actors)’, identified by other authors (Stephens et al. 2018, p. 161).

Industry-affiliated researchers appear frequently in the media. Many of these individuals hold senior positions in starts-ups, such as Mosa Meat or Memphis Meats, which are still pursuing funding and partnerships. Others work for large established meat companies now investing in cultured meat. This means that much of the first-hand insight into the actual state of cultured meat is not coming from independent academics, which may be a function of the lack of publicly funded research on cultured meat (Dolgin 2019). It comes rather from industry-affiliated researchers, who may have strong financial incentives to promote their research. This could in part explain the overwhelmingly positive coverage noted above.

It is also of note that the ‘uncertainty narrative’ only appeared in 4% of articles, in contrast to the sentiment present in the IPCC 2019 report (IPCC 2019). Research has shown that in other areas of science coverage such as climate change, journalists can find the reporting of uncertainties problematic (Painter 2013; Post and Maier 2016), but in this instance, it is the speculative nature of the current scientific and industry reports on cultured meat that is often not sufficiently emphasized. This is probably compounded by the unwillingness of companies to share true representations of the limitations of their technology for fear of scaring away investors.

We found an absence of much discussion about the winners and losers at the production level (Stephens et al. 2018), particularly of the risks of a few high-tech companies based in the Global North playing an exaggerated role in the future global food system and security (Hocquette 2016; Sexton et al. 2019), nor of the issue of which income groups may have access at the consumer level (Pluhar 2010), nor of the issue of whether governments should have a role in facilitating a more just transition to cultured meat production and consumption.

Our results update and confirm the previous (limited) research that media outlets have tended to give a largely positive treatment to cultured meat, and have quoted few oppositional sources including those of the livestock sector which stands to lose the most. However, our research offers much more detailed analysis of the period after 2013 (the most recent period to be covered systematically by previous studies), in which there has been a growth in investor interest, a significant increase in the number of companies in the cultured meat sector, fast changes in technology, and a growing pushback from interest groups.

In particular, we see a greater presence of the US cattle industry responding to the regulatory issues. We have also mapped in much more detail the presence of the type of counter narratives or argumentation that they and others put forward in the media, and the relative weighting of the presence of these and the positive narratives articulated by industry sectors. There are significant overlaps between the narratives in the media and those identified by other methods (Sexton et al. 2019) but interesting differences and additions too, as outlined in the “Results and discussion” section. Aspects of the two dominant metaphors found by Broad (2020) are also present in media narratives, but not to the same level of complexity and detail.

One of the limitations of this study is that the period of media analysis finished before the publication of key reports discussing the cultured meat sector, including the IPCC report on climate change and land (IPCC 2019). However, the inclusion of these reports would not likely have changed the finding on sentiment analysis. For example, the Guardian considered a report written by the consultancy firm Kearney, which suggested that ‘most “meat” in 2040 will not come from dead animals’ (Kearney 2019), to be sufficiently important to place it on the front page of its print edition on 12 June 2019. This bold prediction could of course be questioned, but the same report was also featured with little interrogation of its claims in the Telegraph, the Independent, and the Mail.

Other limitations of our methods include the lack of analysis of coverage on television or social media, or of the media in other countries, such as Holland, Israel, or Spain where important companies working in this sector are based. Given the importance of visual representations on social media (Painter et al. 2017), it would also be helpful to build on the work of Stephens and Ruivenkamp (2016) to examine the images of cultured meat on different media platforms, and how they land with different audiences.

Finally, research has shown that media representations play a role in shaping public attitudes to emerging technologies, and this media influence, although complex, means there are risks of overselling both the benefits and perils of new technologies. On one hand, overestimation of the promise of new technologies can undercut rigorous consideration of the true costs of implementing new technologies. On the other, public sentiment toward genetically modified organisms (GMOs) demonstrates that the public can be turned off by novel technological interventions in the food system, no matter their promised benefits, especially if the technological innovators are seen as untrustworthy profit-seekers (Poortinga and Pidgeon 2007). Similarly, populist-tinged suspicion of global institutions, including large companies, animates growing conspiracy theories involving technologies such as vaccines and 5G infrastructures (Żuk et al. 2019).

Our findings support the previous scholarship on emerging technologies which concluded that with important variations, media treatments are largely positive. The industry dominance in news coverage of cultured meat found here is a concern. On one hand, it fails to provide the public with a realistic account of the current capabilities of this emerging technology. On the other, it may have unintended consequences on public sentiment if cultured meat is slow to realize its promise or if the public begins to lose trust that start-ups and established meat companies will protect consumers’ interests and produce a healthy and safe product.

## Footnotes

1 There is considerable debate about the correct nomenclature (Stephens, Sexton, and Driessen, 2019). For example, the term ‘clean meat’ is frequently used. But this term has at times included plant-based alternatives to meat based on such ‘natural’ products as pea protein, seitan, or soy, which we are not examining in this article. We choose the phrase ‘cultured meat’, while recognizing that other terms are used.

2 https://www.theguardian.com/science/video/2013/aug/05/google-burger-sergey-brin-lab-grown-hamburger

3 https://www.forbes.com/sites/michaelpellmanrowland/2020/01/22/memphis-meats-raises-161-million-series-b-funding-round-aims-to-bring-cell-based-products-to-consumers-for-the-first-time/#10fbb265428d

4 https://www.idtechex.com/en/research-article/what-will-happen-to-the-cultured-meat-industry-in-2020/19210

5 Hereafter, the figures that appear after the percentages represent the number of articles.

6 She is one of the few academic specialists in the field to have been given her own column piece (Observer, 22 January 2012), although this was outside of our sample period.

## References

[CR1] Anderson AA, Scheufele DA, Brossard D, Corley EA (2011). The role of media and deference to scientific authority in cultivating trust in sources of information about emerging technologies. Int J Public Opin Res.

[CR2] Arceneaux N, Schmitz Weiss A (2010). Seems stupid until you try it: press coverage of Twitter, 2006-9. New Media Soc.

[CR3] Bauer MW, Kohring M, Allansdottir A, Gutteling J, Gaskell G, Bauer MW (2001). The dramatisation of biotechnology in the elite mass media. Biotechnology 1996–2000: the years of controversy.

[CR4] Bhat ZF, Kumar S, Fayaz H (2015) In vitro meat production: Challenges and benefits over conventional meat production. J Integr Agric 14(2):241–248

[CR5] Brennen JS, Howard PN, Kleis Nielsen R (2018) An Industry-Led Debate: How UK Media Cover Artificial Intelligence. Reuters Institute for the Study of Journalism, Oxford, UK

[CR6] Broad GM (2020) Making meat better: the metaphors of plant-based and cell-based meat innovation. Environ Commun 1-14

[CR7] Bryant C, Barnett J (2018). Consumer acceptance of cultured meat: a systematic review. Meat Sci.

[CR8] Bryant C, Barnett J (2019). What’s in a name? Consumer perceptions of in vitro meat under different names. Appetite.

[CR9] Cameron B, O’Neill S (2019) State of the industry report: cell-based meat. Washington DC: the Good Food Institute

[CR10] Caulfield T (2004). Biotechnology and the popular press: hype and the selling of science. Trends Biotechnol.

[CR11] Cogan B (2005). “Framing usefulness:” an examination of journalistic coverage of the personal computer from 1982–1984. South Communic J.

[CR12] Czitrom DJ (1982) Media and the American mind: from Morse to McLuhan. Univ of North Carolina Press

[CR13] Datar I, Betti M (2010). Possibilities for an in vitro meat production system. Innov Food Sci Emerg Technol.

[CR14] Dilworth T, McGregor A (2015). Moral steaks? Ethical discourses of in vitro meat in academia and Australia. J Agric Environ Ethics.

[CR15] Dolgin E (2019) Sizzling interest in lab-grown meat belies lack of basic research. Nature News (6 February) 10.1038/d41586-019-00373-w 30755743

[CR16] Donk A, Metag J, Kohring M, Marcinkowski F (2012). Framing emerging technologies: risk perceptions of nanotechnology in the German press. Sci Commun.

[CR17] Dudo A, Dunwoody S, Scheufele DA (2011). The emergence of nano news: tracking thematic trends and changes in U.S. newspaper coverage of nanotechnology. J Mass Commun Q.

[CR18] Froggatt A, Wellesley L (2019). Meat analogues: considerations for the EU.

[CR19] Goodwin JN, Shoulders CW (2013). The future of meat: a qualitative analysis of cultured meat media coverage. Meat Sci.

[CR20] Happer C, Philo G (2013). The role of the media in the construction of public belief and social change. J Soc Polit Psychol.

[CR21] Hocquette J-F (2016) Is in vitro meat the solution for the future? Meat Sci 120:167–176. 10.3389/fsufs.2019.00045 10.1016/j.meatsci.2016.04.036 27211873

[CR22] Hopkins P, Dacey A (2008). Vegetarian meat: could technology save animals and satisfy meat eaters?. J Agric Ethics.

[CR23] Hopkins P (2015). Cultured meat in western media: the disproportionate coverage of vegetarian reactions, demographic realities, and implications for cultured meat marketing. J Integr Agric.

[CR24] International Livestock Research Institute (ILRI) (2019) Options for the livestock sector in developing and emerging economies to 2030 and beyond. Retrieved from https://www.ilri.org/publications/options-livestock-sector-developing-and-emerging-economies-2030-and-beyond

[CR25] Intergovernmental Panel on Climate Change (IPCC), (2019) Climate change and land, summary for policy makers. Geneva, Switzerland: IPCC

[CR26] Kearney AT (2019). How will cultured meat and alternative meat products disrupt the agricultural and food industry?.

[CR27] Kitzinger J, Williams C (2005). Forecasting science futures: Legitimising hope and calming fears in the embryo stem cell debate. Soc Sci Med.

[CR28] Lee C, Scheufele D, Lewenstein B (2005). Public attitudes toward emerging technologies: examining the interactive effects of cognitions and affect on public attitudes toward nanotechnology. Sci Commun.

[CR29] Lynch J, Pierrehumbert R (2019) Climate impacts of cultured meat and beef cattle. Front Sustain Food *Syst*:3(5). 10.3389/fsufs.2019.00005 10.3389/fsufs.2019.00005 PMC6751088 31535087

[CR30] Marks LA, Kalaitzandonakes N, Allison K, Zakharova L, Santaniello V, Evenson RE, Zilberman D (2002). Time series analysis of risk frames in media communication of agrobiotechnology. Market developments and genetically modified foods.

[CR31] Mattick CS, Landis AE, Allenby BR (2015). A case for systemic environmental analysis of cultured meat. J Integr Agric.

[CR32] Mattick CS, Landis AE, Allenby BR, Genovese NJ (2015). Anticipatory life cycle analysis of *in vitro* biomass cultivation for cultured meat production in the United States. Environ Sci Technol.

[CR33] McHugh ML (2012). Interrater reliability: the kappa statistic. Biochemia Medica (Zagreb).

[CR34] Metag J, Marcinkowski F (2014). Technophobia towards emerging technologies? A comparative analysis of the media coverage of nanotechnology in Austria, Switzerland and Germany. Journalism.

[CR35] Newman N, Fletcher R, Kalogeropoulos A, Nielsen RK (2019). Digital news report.

[CR36] Nisbet MC, Lewenstein BV (2002). Biotechnology and the American media: the policy process and the elite press, 1970 to 1999. Sci Commun.

[CR37] O’Neill S, Williams HTP, Kurz T, Wiersma B, Boykoff M (2015). Dominant frames in legacy and social media coverage of the IPCC Fifth Assessment Report. Nat Clim Chang.

[CR38] Painter J (2013) Climate Change in the Media: Reporting Risk and Uncertainty. IB Tauris

[CR39] Painter J et al. (2017) Something Old, Something New: Digital Media and the Coverage of Climate Change. Reuters Institute for the Study of Journalism, Oxford, UK

[CR40] Pluhar EB (2010). Meat and morality: alternatives to factory farming. J Agric Environ Ethics.

[CR41] Poortinga W, Pidgeon J, Brossard D, Shanahan J, Nesbitt TC (2007). Public perceptions of agricultural biotechnology in the UK: the case of GM foods. The media, the public and agricultural biotechnology.

[CR42] Post MJ (2012). Cultured meat from stem cells: challenges and prospects. Meat Sci.

[CR43] Post S, Maier M (2016). Stakeholders’ rationales for representing uncertainties of biotechnological research. Public Underst Sci.

[CR44] Priest SH (2008). North American audiences for news of emerging technologies: Canadian and US responses to bio- and nanotechnologies. J Risk Res.

[CR45] Schaefer GO, Savulescu J (2014). The ethics of producing *in vitro* meat. J Appl Philos.

[CR46] Schmidt A, Schäfer MS, Ivanova A (2013) Media Attention for Climate Change around the World: A Comparative Analysis of Newspaper Coverage in 27 Countries. Glob Environ Change 23(5):1233–1248.

[CR47] Schneider Z (2013). In vitro meat: space travel, cannibalism, and federal regulation. Houst Law Rev.

[CR48] Scheufele DA, Lewenstein BV (2005). The public and nanotechnology: how citizens make sense of emerging technologies. J Nanopart Res.

[CR49] Sexton AE, Garnett T, Lorimer J (2019) Framing the future of food: the contested promises of alternative proteins. Environ Plan E: Nat Space 2(1) 10.1177/2514848619827009 PMC6989034 32039343

[CR50] Sharma S, Thind SS, Kaur A (2015). In vitro meat production system: why and how?. J Food Sci Technol 2015.

[CR51] Stephens N, Ruivenkamp M (2016) Promise and ontological ambiguity in the *in vitro* meat imagescape: from laboratory myotubes to the cultured burger Sci Cult 25(3) 10.1080/09505431.2016.1171836 PMC5022697 27695202

[CR52] Stephens N, Dunsford I, Di Silvio L, Ellis M, Glencross A, Sexton A (2018). Bringing cultured meat to market: technical, socio-political, and regulatory challenges in cellular agriculture. Trends Food Sci Technol.

[CR53] Stephens N, Sexton A, Driessen C (2019) Making sense of making meat: key moments in the first 20 years of tissue engineering muscle to make food. Front Sustain Food Syst. 10.3389/fsufs.2019.00045 10.3389/fsufs.2019.00045 PMC7611147 34250447

[CR54] Tuomisto HL (2018) The eco-friendly burger: could cultured meat improve the environmental sustainability of meat products? Sci Soc. 10.15252/embr.201847395 10.15252/embr.201847395 PMC6322360 30552146

[CR55] Tuomisto HL, de Mattos M (2011). Environmental impacts of cultured meat production. Environ Sci Technol.

[CR56] Vilella-Vila M, Costa-Font J (2008). Press media reporting effects on risk perceptions and attitudes towards genetically modified (GM) food. J Socio-Econ.

[CR57] World Economic Forum (WEF) (2019) Meat: the future series; alternative proteins. Geneva, Switzerland: World Economic Forum. Retrieved from http://www3.weforum.org/docs/WEF_White_Paper_Alternative_Proteins.pdf

[CR58] Zaraska M (2016) Meatballs made in a lab: ick or slick? *Washington Post*. 3 May

[CR60] Żuk P, Żuk P, Lisiewicz-Jakubaszko J (2019). The anti-vaccine movement in Poland: the socio-cultural conditions of the opposition to vaccination and threats to public health. Vaccine.

